# Current Perspectives on Adult Mesenchymal Stromal Cell-Derived Extracellular Vesicles: Biological Features and Clinical Indications

**DOI:** 10.3390/biomedicines10112822

**Published:** 2022-11-05

**Authors:** Giusi Alberti, Eleonora Russo, Simona Corrao, Rita Anzalone, Peter Kruzliak, Vitale Miceli, Pier Giulio Conaldi, Francesca Di Gaudio, Giampiero La Rocca

**Affiliations:** 1 Department of Biomedicine, Neurosciences and Advanced Diagnostics (BiND), University of Palermo, 90127 Palermo, Italy; 2 Department of Surgical, Oncological and Stomatological Sciences (DICHIRONS), University of Palermo, 90127 Palermo, Italy; 3 Department of Medical Biology, Jessenius Faculty of Medicine, Comenius University in Bratislava, 03601 Martin, Slovakia; 4 Research Department, IRCCS ISMETT (Istituto Mediterraneo per i Trapianti e Terapie ad Alta Specializzazione), 90127 Palermo, Italy; 5 PROMISE Department, University of Palermo, 90127 Palermo, Italy

**Keywords:** adult mesenchymal stromal cells, bone marrow, adipose tissue, extracellular vesicles, inflammation, tissue repair, regeneration, cancer, cell-free therapies

## Abstract

Extracellular vesicles (EVs) constitute one of the main mechanisms by which cells communicate with the surrounding tissue or at distance. Vesicle secretion is featured by most cell types, and adult mesenchymal stromal cells (MSCs) of different tissue origins have shown the ability to produce them. In recent years, several reports disclosed the molecular composition and suggested clinical indications for EVs derived from adult MSCs. The parental cells were already known for their roles in different disease settings in regulating inflammation, immune modulation, or transdifferentiation to promote cell repopulation. Interestingly, most reports also suggested that part of the properties of parental cells were maintained by isolated EV populations. This review analyzes the recent development in the field of cell-free therapies, focusing on several adult tissues as a source of MSC-derived EVs and the available clinical data from in vivo models.

## 1. Introduction

The first evidence of the existence of fibroblastoid cells, in the bone marrow (BM) of mice, capable of forming colonies from a single cell (the colony-forming unit fibroblast, CFU-F) was obtained in 1970 by Friedenstein and coworkers. In particular, they demonstrated the ability of these cells to reconstitute the hematopoietic microenvironment and also to regenerate bone tissue in serial transplantation experiments [1,2]. It was in 1998 that Owen and Friedenstein further confirmed the self-renewal and plasticity capabilities of these stromal cells [3]. Caplan and colleagues, exploring the mechanisms underlying osteogenic and chondrogenic differentiation, using mesenchymal cells of limb buds from chick embryos, first coined the term mesenchymal stem cells (MSCs) (reviewed in [4]). Moreover, by means of in vitro and in vivo studies, they reported that MSCs residing in the BM, periosteum, and connective tissue should be cultured and differentiated into bone and cartilage [4]. Subsequently, Pittenger et al. showed that human BM contains a subpopulation of stromal cells with the ability to differentiate in vitro into a variety of mesenchymal-derived lineages (such as osteoblasts, chondrocytes, and adipocytes) [5,6,7]. It is becoming increasingly clear that these cells may represent a therapeutic strategy due to their multilineage differentiation capabilities, alongside anti-inflammatory, immunomodulatory, regenerative, and antimicrobial properties. This is further confirmed by their capacity for homing and migration to lesion sites, and the safety profile in allogeneic transplantation [8,9,10,11]. In 2005, the International Society for Cellular Therapy (ISCT) recommended using the nomenclature of “mesenchymal stromal cells (MSCs)” to describe stromal progenitors found in multiple tissue types, which can be induced to differentiate in vitro into different lineages beyond skeletal tissues [12]. Indeed, the sources of MSCs are divided into three categories according to the site/time of collection: (i) adult sources, such as bone marrow, adipose tissue, dental pulp, and peripheral blood; (ii) embryonic sources, such as embryonic tissues; (iii) perinatal-derived sources, as the placenta and umbilical cord. Adult MSCs can be successfully isolated from a number of adult organs since they occupy specific niches within them. These cells feature a striking differentiative potency and promising therapeutic effects [13,14]. There are many ways in which MSCs can ameliorate an underlying pathological condition; examples include matrix remodeling and the production of diffusible factors, including cytokines, hormones, and extracellular vesicles (EVs) [14,15,16]. These chemical messengers are able to inhibit apoptosis, stimulate proliferation, promote vascularization, and modulate the immune response, showing the ability to induce cellular changes in nearby cells [14,17,18,19].

EVs (such as exosomes and microvesicles) are constituted by a variety of molecular species, which are encapsulated into a membrane. Cells secrete EVs in the pericellular space, where they act as cell–cell communication devices both in physiology and in diseases [20,21]. The content of EVs, biologically active, may therefore reflect the features of the parental cells, as well as the local conditions in the tissue. Consequently, it has been proposed that EVs can be directly utilized as alternatives to intact cells in a cell-free manner for therapeutic purposes; indeed, they confer some advantages, such as the reduction in the risk of immune rejection and tumorigenicity deriving from cell transplantation [18]. Recently, an increasing number of reports have confirmed the therapeutic impact of EVs from different adult MSC sources on various diseases, such as immune and inflammatory disorders and cancers [22,23,24].

In this review, we summarize the recent findings in the literature regarding the novel applications of EVs derived from different populations of adult mesenchymal stromal cells (MSC-derived EVs (MSC-EVs)) in terms of clinical usefulness in various diseases. Finally, we use nomenclature in accordance with the recommendations of the International Society for Extracellular Vesicles (ISEV) stating that: “EV is the preferred generic term for the subject of our investigations” [25].

## 2. Extracellular Vesicles

Intercellular communication is a fundamental process of living organisms. This can be carried out by several means, and one of the most intriguing mechanisms used by cells to communicate, locally or at a distance, is through EVs. They are small spherical membrane vesicles with a phospholipid bilayer, with a content that includes specific molecular species which were originally present in either the cytoplasm or the membrane of the parental cell [26]. Since the initial isolation of EVs from blood cells, the studies on EVs demonstrated a striking variability in terms of cellular origin, molecular content, size, and efficacy [27]. Recent findings have demonstrated that the secretion of the vesicles is a well-conserved mechanism, common to the majority of the cells (both prokaryotic and eukaryotic), and describes vesicle entities released by living cells through a secretory pathway during physiological and pathological processes [28]. Despite their first classification on the basis of their size (e.g., apoptotic bodies, 1–5 µm; microparticles, 0.1–1 µm; exosomes, 30–150 nm), alternative classifications have been proposed (partially overlapping the first classification): for some EVs, the tissue of origin has been considered as a means of classification (e.g., prostasomes, oncosomes); in other instances, functional and other parameters have been considered [29]. Apart from the classification issues, the main problem which can be found in literature is the lack of a consensus on which molecules can be used as unique references (markers) for their classification into different vesicular subgroups (i.e., micro- or nanovesicles). As a consequence, allocating a vesicle to a restricted origin pathway is possible only if live imaging techniques are used. In this case, it is possible to make a strong association between the investigated vesicle and its biogenesis in the parental cell [30]. An attempt to solve the classification issue was made by scientists of ISEV, who have drawn up the latest document on the “minimum information to be used for studies on extracellular vesicles 2018” (MISEV2018), which recommended that the term “extracellular vesicle” should be used as a formal description for these vesicles. Specifically, they suggested sorting vesicles based on various features which can globally define the EV type and feature. Briefly, an EV can be described by its size and density and the presence of distinctive molecular species (e.g., CD63, CD81, Annexin 5). Other proposed features are tissue origin as well as biogenesis and function [25].

### 2.1. Biochemical Composition

The molecular composition of EVs depends on the vesicle types, as well as the cells from which they are derived, and is dependent on the status of the parental cell type [21], or specific conditions [31]. The main components of the EVs are proteins and lipids, as well as nucleic acids (largely constituted by mRNAs and miRNAs) [32]. However, the nature of the EV subtypes is also a key factor in determining the molecular contents in EVs [33]. EV proteins reflect both the cellular origin and the content of the originating compartments; therefore, one important group is represented by integral membrane proteins (tetraspanins such as CD9, CD63, and CD81). Moreover, organellar proteins may be present but usually not in exosomes. The presence of cytoplasmatic molecules usually reflects the biogenetic pathway that originated the specific EVs (as for some Hsps). In some instances, molecules that are normally extracellular may also be present, as demonstrated for acetylcholinesterase (ACHE). In addition, membrane and cytoskeletal proteins, lysosomal enzymes, cytokines, antigen presentation-related proteins (major histocompatibility complex (MHC) class I and II/peptide complexes), transcription and protein synthesis-related proteins, some heat shock proteins (Hsps), and other specific proteins can be also part of these vesicles [30]. Importantly, all of these molecules can have functional effects on the recipient cells, representing an important factor in cell–cell interactions. Moreover, membrane proteins can be used as specific biomarkers of diseases as they may reflect pathological conditions in the source cells [34].

EV lipid composition is the least known parameter, probably due to the few standardized isolation methods currently used. Despite these limitations, it is known that EVs bear molecules (lipids) usually found in plasmatic membranes [35]. Moreover, it has been demonstrated that EVs of different diameters are made up of different lipids [36].

EVs also contain RNA molecules that can be transferred into recipient cells, where the mRNAs can be translated into proteins, resulting in an alteration of target cell behavior [37]. Of note, the RNA component of EVs is of particular diagnostic interest, as naked RNAs are not stable in blood outside of the vesicles, due to exposure to RNases. The classes of RNAs most abundant in EVs are microRNAs (miRNAs) and messenger RNAs (mRNAs), together with long noncoding RNAs (lncRNAs), piwi-interacting RNAs (piRNAs), and circular RNAs (circRNAs) [38,39].

The existence of DNA in EVs has been revealed within the past decade [40]. The first studies focused on double-stranded DNA within EVs from cell line conditioned media, thus suggesting it as a true vesicular biomarker [41]. It is likely that distinct types (single-stranded, double-stranded, mitochondrial) of DNA exist in EVs. Moreover, DNA may be present in different forms (fragment length, chromatin-bound) that may further help discriminate the distinct type of EVs based on their cell of origin [42]. However, it is not yet clear why and how cells communicate via EV-DNA and what the functional role of this communication is, just as there are no validated and standardized protocols for the study of this biological feature.

### 2.2. Biogenesis and EV Diversity

The biogenesis of EVs is a mechanism that differs among various types of vesicles. For instance, secretion of apoptotic bodies (>800 nm) occurs during apoptosis, a process allowing undesired cells that can alter physiological homeostasis to be discarded [43]. Apoptosis is mainly divided into three steps, with the final formation of membrane protrusions that regulate cell fragmentation and release in the form of apoptotic bodies [44]. Apoptotic blebbing is monitored by multiple events (for example, hydrostatic pressure and contraction) which promote the translocation of fluids and cell components into these newly forming membrane blebs. Although the mechanisms of apoptotic body formation are still not well understood, apoptotic bodies seem to play an important role in intercellular communication since they transport specific biomolecules to neighboring cells; for instance, apoptotic bodies coming from cancerous processes can deliver altered molecules to other recipient cells, stimulating the proliferation of new cancer cells and the acceleration of metastatic processes [45].

Microvesicles (MVs), also known as shedding vesicles, microparticles, or ectosomes, are particles with a diameter range of 0.1–1 µm, originating from the plasma membrane of the cell through a process of outward budding [46]. Specifically, the increase in intercellular Ca^++^, induced by an external signal, causes changes in the lipid distribution of the bilayer and the concomitant membrane blebbing, also due to the activity alteration of the phospholipid transportation enzymes [47]. Calcium flux has been associated with a remodeling of cytoskeletal proteins, which is believed to be responsible for the membrane plasticity leading to the detachment of EVs [48].

Exosomes are the most studied vesicles, mostly defined by their size and their protein content, despite the fact that in literature the term “exosome” is improperly used to refer to small EVs. They are vesicles of 30–150 nm, forming through a well-organized process that starts with the invagination of the plasma membrane of the parental cell and the development of an endocytic pathway [49]. Cell endocytosis is a molecular mechanism that develops during degradation, recycling, signaling, cytokinesis, and migration processes. It occurs when the plasma membrane introflects towards the center of the cell and forms small vesicles named “endosomes” [49]. Endosomal maturation is a deeply regulated process, where proteins and lipids (Rab, GTPases, and phosphoinositides (PIPs)) work in harmony with the endoplasmic reticulum (ER) and the trans-Golgi network (TGN), providing enzymes and assisting the exchange of membrane components [50]. When a new endosome fuses with a previously formed early endosome (EE), the latter matures into a sorting endosome that is converted into a late endosome (LE) carrying cargo that needs to be destroyed by the lysosomal system. The degradation of its content starts with the development of intraluminal vesicles (ILVs) in the EE. Once LEs/multivesicular bodies (MVBs) are formed, the majority of them undergo a degradation pathway fusing with lysosomes, whereas a few escape from this process, fusing with the plasma membrane and releasing their ILVs in the form of exosomes [51]. Finally, the development of the MVBs is regulated by the activity of the endosomal sorting complex required for transport (ESCRT), which is composed of approximately 30 proteins that assemble into four complexes (ESCRT-0, -I, -II, and -III) with associated proteins (VPS4, VTA1, and ALIX (also called PDCD6IP)) conserved from yeast to mammals [52]. ALIX and other exosomal markers (i.e., CD63 and Hsp70) are important for many of the signaling events occurring during the formation of ILVs and exosomes [53]. Various populations of ILVs, with different lipids and cargo, can cohabit and form within MVBs and can be distinguished based on their size and their mechanism of formation. However, their mechanisms of formation and sorting still remain partially understood, with the complex ESCRT process being the best described so far; nonetheless, some other ESCRT-independent pathways have also been identified and might coexist with ESCRT-dependent machinery, thus confirming the presence of different MVB subpopulations [54].

Currently, isolation and characterization of EVs are performed by using different methods to create an experimental pathway to obtain the desired population. In particular, characterizing EVs may require one or more of different approaches, such as electron microscopy (TEM, SEM, CrioTEM), protein-specific analyses (Western blot, ELISA), -omics approaches (proteomic, lipidomic, metabolomic), dynamic light scattering techniques (nanoparticle tracking analysis (NTA) and Zetasizer), and tunable resistive pulse sensing (TRPS). In addition, other groups focused on the use of immunocytochemical and flow cytometric analyses of specific EVs markers [55]. Each combination of methods (or single method if applicable) obviously presents some positive points and some negative aspects, leading at the end to the purification of a different population of vesicles, even if starting from the same biological material. Therefore, at the moment, there is no single standardized method for the isolation of EVs, which could be positively reflected in less variability and allowing the comparison of scientific results, even when the starting biological material is the same [56]. Literature analysis indicated that there are disparate methods for vesicle preparation, such as differential centrifugation/ultracentrifugation coupled with sucrose/iodixanol density gradient, size exclusion chromatography, and specialized precipitation-based methods [56]. At the moment, while all these methods present advantages and pitfalls, none of them has emerged as a gold standard. It is apparent that a combination of different isolation, separation, and characterization techniques may result in the optimal mix to obtain EVs of high quality, both in terms of numbers and quality, which are the essential features needed for downstream applications. In this regard, immunoaffinity methods may be highly advantageous for the selective isolation of EVs starting from different cell samples [57]. Furthermore, limitations associated with the use of the common methods could be bypassed with the use of microfluidic platforms that use different isolation principles (e.g., they allow for large volumes of starting samples). Despite the wide range of isolation methods currently available, the scientific community is still arguing about the choice of the best protocol to use, since each may be suitable for the analysis of one type of sample, rather than another. Hence, in absence of a precise standardization, the choice of the isolation method should follow the characteristics of the sample that has to be analyzed.

### 2.3. EV Functions

EVs are part of the cell-to-cell communication network that regulates physiological and pathological processes in all living organisms [58]. EVs play an important role in several physiological conditions, such as coagulation [59], pregnancy [60], metabolism [61], immunity [62], and apoptosis [50]. In stress conditions (e.g., induced by ionizing radiation, osmotic alterations, hypoxia, and other stimuli), EVs act as messengers of stress response towards near and distant cells [63]. Stress events affect not only the amount of secreted EVs, but also their molecular composition, resulting in altered vesicles that can prospectively provide biomarkers for several diseases [64]. Indeed, they might act as reservoirs of dangerous molecules (e.g., unfolded proteins, mutated DNA, or pathogens) that could modify cell physiology and thus need to be eliminated through normal clearance [65]. For instance, it has been proposed that when the normal activity of the endoplasmic reticulum fails, due to stress events, cells release stressed EVs that trap damaged/unfolded proteins inside their lumen in order to preserve the endoplasmic reticulum homeostasis and cell vitality [66]. Another interpretation of “stressed” EVs describes these vesicles as alarm bells that warn cells, and the immune system, of possible dangers caused by stressful events [67]. For these reasons, EVs are considered key actors in several diseases, and the role of EVs in the development of some neurodegenerative diseases is of particular interest [68]. During inflammation and brain damage events, EVs cross the blood–brain barrier and regulate communication, in both directions, between the central nervous system and the periphery. This vesicle type carries altered and aggregated proteins (e.g., beta-amyloid or prion proteins) that are associated with some neurodegenerative diseases [69]. EVs may be also implicated in some blood disorders. For example, platelet EVs (P-EVs) play several roles in the activation of the immune system through the interaction with leukocytes, in atherosclerotic lesion formation, and in coagulation processes [70,71]. Another important role of EVs has been also shown in several metabolic processes, affecting serum metabolome modifications in different areas of the organism [72]. For instance, it has been demonstrated that high levels of EVs rich in arginase are involved in the conversion of arginine into ornithine, causing anomalies in the process of vascularization, neuronal toxicity, and cancer [73]. However, these alterations are not limited to the activity of the liver alone; stem cells, red blood cells, and endothelial cells can also release enzymes within EVs that may increase the risk of pathological conditions (e.g., high blood pressure, elevated glucose and triglyceride levels) and related disorders (e.g., type 2 diabetes, dyslipidemia, obesity) [74,75,76]. In adipose tissue (AD), high levels of EVs may be responsible for different pathological conditions (i.e., insulin resistance and secretion of proinflammatory adipokines) and chronic inflammation [77]. In cancer, EVs are involved in several features of the disease, especially in the activation of aberrant proliferative and suppressor pathways. Tumor EVs carry specific molecules that may be taken up by other cells and alter the expression of pro- and antiproliferation genes [78]. Moreover, EVs act as intercellular communicators between tumor cells and distant organs during metastatic colonization [78].

For these reasons, the interest in EVs as disease biomarkers has been growing very fast over the years [79], and EVs as disease biomarkers are detectable in almost all types of body fluids, such as blood, urine, saliva, breast milk, and ascites [24]. Currently, information on the proteins, lipids, and RNAs expressed in EVs is collected in VESICLEPEDIA (http://www.microvesicles.org; accessed on 10 August 2022) [80], while the exosomes of different cell types and organisms are described in the ExoCarta database [81]. However, one of the principal obstacles to their clinical application is their actual yield from donor cells, which is often very poor and strongly related to the protocol of isolation [82]. Consequently, there is a need for further investigations to further develop the knowledge of EVs in both physiological and pathological conditions.

## 3. Adult Mesenchymal Stromal Cell-Derived EVs

**Figure 1:**
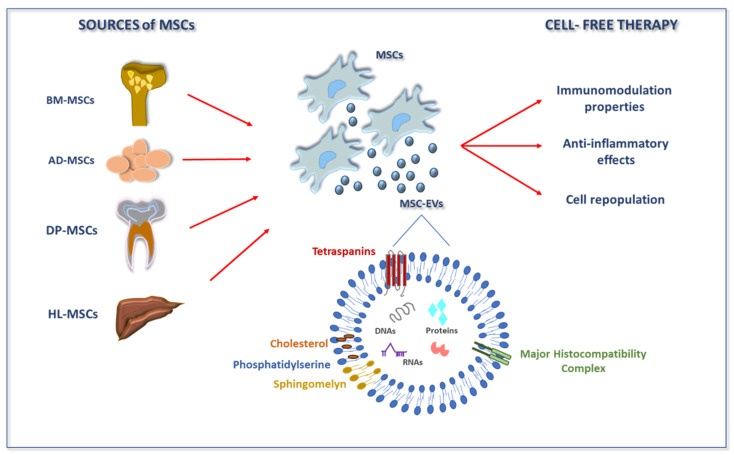
Origin and therapeutic indications of MSCs and MSC-EVs. Several adult tissues have been characterized as sources of MSCs. Under physiological and pathological conditions, MSCs can release EVs into the extracellular space. These vesicles contain a cargo that features various classes of biomolecules, including key signaling proteins, lipids, and nucleic acids. Literature data showed that MSC-EVs can mediate various therapeutic roles, such as immune modulation and anti-inflammatory effects, as well as promoting tissue regeneration. Abbreviations: AD-MSCs, adipose mesenchymal stromal cells; BM-MSCs, bone marrow mesenchymal stromal cells; DP-MSCs, dental pulp mesenchymal stromal cells; EVs, extracellular vesicles; HL-MSCs, human liver mesenchymal stromal cells; MSCs, mesenchymal stromal cells.

Recently, it has been acknowledged that the mechanistic explanation of the effectiveness of MSCs is not only due to their ability to migrate and survive in diseased areas. The role of proper differentiation is also being viewed as less necessary, since recent reports more focus on paracrine signaling. Indeed, MSC-EVs are able to improve the diseased organs in several in vivo models, from myocardial infarction to stroke to kidney injury and others [83,84,85]. MSC-EVs can thus constitute a promising cell therapy approach in which a cell-free mixture of vesicles is used. In addition, relevant studies demonstrated that similarly to parental cells, vesicles did not show toxicity, had low immunogenicity, and remained immunosuppressive [86,87]. It is clear that the features of the MSC populations from which EVs are derived are reflected in the final properties and composition of EVs [88]. The same can be stated when considering the in vitro expansion conditions (e.g., by various preconditioning methods [14]), the harvesting period, and the enrichment methods that affect the structural and functional heterogeneity of EVs. This explains the differences existing between adult MSCs and perinatal MSCs secretomes [89]. Adult MSCs exist in many tissues of the human body, e.g., BM, AD, peripheral blood, dental pulp (DP), and endometrial tissues; therefore, EVs can be released by virtually all of them (Figure 1). Nonetheless, most of the information available regarding EVs is related to the two most common sources of adult MSCs, i.e., BM and AD. Like their parental cells, MSC-EVs have also been studied for their key role in the physiological and pathological processes that affect humans, as they are involved in different cellular processes, such as modulation of angiogenesis, cell proliferation, and immune regulation [87] (Figure 1).

Several groups investigated the protein composition of vesicles derived from adult MSCs. The first study in 2012 allowed the identification of 730 proteins by proteomic analysis of human BM-derived MSC-EVs (BMMSC-EVs). This analysis found that markers belonged to both MSCs and EVs; in addition, the authors identified signaling molecules involved in the self-renewal and differentiation capacities of MSCs, and a number of proteins with key roles in various cellular processes (e.g., cell proliferation, adhesion, migration, and morphogenesis) [90]. In another study by Angulski et al., 797 proteins were identified in human BMMSC-EVs, of which 60% overlapped with those already identified in the previous studies [91]. However, the protein list of BMMSC-EVs also depends on the culture conditions of these cells. Indeed, comparing the proteomic profile of BMMSC-EVs under normoxic and hypoxic conditions showed that out of 1927 total proteins, 457 were present exclusively in hypoxic MSC-EVs [92]. Of note, the hypoxic conditions increase the activation of genes involved in the angiogenic process, such as platelet-derived growth factor, fibroblast growth factor, and plasminogen activator [92]. In a proteomic study on an experimental animal model of rats with subcortical stroke, 2416 proteins from AD-derived MSC-EVs (ADMSC-EVs) were identified. Most of these proteins are associated with brain repair function. These proteins presented hydrolase activity, as well as tubulin binding ability, and kinase activity [93]. Instead, ADMSC-EVs of swine were enriched in proteins involved in tissue regeneration processes generally mediated by MSCs [94]. In addition, the analysis of the proteomic profiles of ADMSC-EVs compared to their parental MSCs of swine with metabolic syndrome and normal controls led to the discovery that some proteins were upregulated only in the control ADMSC-EVs and were mainly associated with tissue regeneration; while the proteins present only in the MSC-EVs derived from swine with metabolic syndrome were linked to proinflammatory pathways [95].

The molecular profile of MSC-EVs includes various RNAs, which in the case of mRNA can be not only transferred to recipient cells but also translated. However, there is a lack of information on the complete RNA content of MSC-EVs, and the variability linked to the derivation of the parental MSC type also remains to be investigated. Recently, a comparative study on the RNA profile of ADMSC-EVs and BMMSC-EVs revealed a substantial similarity; nonetheless, their relative proportions are different. This study focused on the miRNA content of vesicles from the two cell types: while ADMSC-EVs were enriched in miR-486-5p, miR-10a-5p, miR-10b-5p, miR-191-5p, and miR-222-3p, BMMSC-EVs showed an increased relative abundance in miR-143-3p, miR-10b-5p, miR-486-5p, miR-22-3p, and miR-21-5p [96]. In another study, the miRNA profiling analysis of human BMMSC-EVs revealed that they are involved in several processes and pathways, such as angiogenesis, cellular proliferation, and apoptosis [97]. A transcriptomic study of swine ADMSC-EVs found 4 miRNAs and 255 mRNAs that were particularly enriched in EVs compared to MSCs [98].

Among EV contents, the presence and function of lipid cargo are almost unknown. This is probably due to the difficulty of analyzing these extremely regulated molecules within EVs with specific protocols. A comparative high-resolution lipidomic analysis of MVs and exosomes derived from BM-MSCs showed an enrichment in exosomes for glycolipid, free fatty acids, phosphatidylserine, and cardiolipin, while lysoderivatives of phosphatidylserine, phosphatidylglycerol, and phosphatidylinositol were abundant in both MVs and exosomes [99].

It is clear that further studies are needed to better clarify the full range of molecular species that can be found in EVs generated by adult MSCs, as well as their mechanistic roles in the different testing environments.

## 4. Features and Applications of EVs from Adult MSCs

A number of studies showed that EVs derived from adult MSCs were accountable for the therapeutic effects of MSCs, emerging as potent components in the “secretory pathways” [88]. Adult MSC-EVs are able to mimic stem cell properties [100], present a decreased malignant potential, and are less immunogenic than the origin cells [101]. As we mentioned above, MSC-EVs are wrapped in a lipid membrane enriched by a number of molecules that serve as markers. Regarding the specific molecules that can identify the molecular signature of vesicles, it has been reported that besides consistent exosomal molecules such as CD9 and CD81, MSC-EVs maintain the expression on their surface of characteristic MSCs markers such as CD29, CD44, and CD73. On the other hand, MSC-EVs lack expression of definitive hematopoietic lineage markers (i.e., CD34, CD14, CD45, CD11b, CD19, and HLA-DR), explaining in part their low immunogenicity [90,102].

### 4.1. Bone Marrow MSC-Derived EVs

The extensive investigations on BM-MSCs allowed the in-depth characterization of these cells with respect to alternative sources. Nonetheless, the isolation requires an invasive procedure with a risk of complications, and also the cellular yield from the BM decreases with the aging of the donor [103,104]. Specifically, Sacchetti et al. provided evidence to confirm that BM-MSCs constitute a subset of perivascular/endothelial CD146+ cells with self-renewal capabilities in in vivo experiments [105]. However, the therapeutic benefits of BM-MSCs are mostly attributable to their secretome, including EVs. Accordingly, we discuss in the following section the therapeutic potential of BMMSC-EVs in several disease settings.

The role of EVs in osteoarthritis has been highlighted by studies showing that BMMSC-EVs actively stimulated resident chondrocytes to reactivate the secretion of several ECM molecules, such as proteoglycans and collagens [106]. The same report also highlighted the role of EVs on the underlying inflammatory process, in that EVs were able to reduce it [106] (Table 1).

Renal injury is another emerging clinical indication for the use of BMMSC-EVs. For instance, considering glycerol-induced acute kidney injury, researchers showed that mice underwent a recovery that was proved both in morphology and kidney functionality following the administration of BMMSC-EVs [107] (Table 1). In another experiment of renal ischemia–reperfusion injury induced by ATP depletion, the cargo in miRNAs of BMMSC-EVs modulated the gene expression of molecules involved in the apoptotic process, hypoxia, and cellular processes such as cytoskeleton organization. The overall result was a protective effect towards kidney cells [108] (Table 1). Furthermore, the overexpression of the miR-let7 load of BMMSC-EVs in the kidney attenuated fibrosis in vivo and reduced TGF-β1-stimulated renal cell damage in vitro in a mouse model of unilateral ureteral obstruction [109] (Table 1). Another approach where BMMSC-EVs can be effective is in the therapy of diabetic nephropathy. Authors demonstrated that vesicles induced an amelioration of the fibrotic process, which was corroborated by the change in the expression levels of molecules that are known to act in the fibrogenic process [110] (Table 1).

With regard to anti-inflammation, BMMSC-EVs play different roles. For instance, BMMSC-EVs were able to promote the development of an anti-inflammatory phenotype in human regulatory macrophages (Mregs) and amplified pro-resolving properties, in connection with the decrease in levels of key molecules such as IL-22 and -23 [111] (Table 1). With regard to spinal cord injury, BMMSC-EVs were shown to reduce the apoptosis and the production of proinflammatory cytokines by inhibiting PTEN and NF-κB signaling via miR-181, both in in vitro and in vivo studies [112] (Table 1). Tendons are other organs in which the specialized connective tissue present can benefit from treatments with BMMSC-EVs. The roles that have been exploited for these EVs range from a direct healing effect, due to the reduction in the apoptotic and inflammatory processes, to the increase in the local concentration of progenitor cells [113] (Table 1). Many studies have also examined the potential applications of BMMSC-EVs in graft-versus-host disease (GVHD) amelioration. It has been observed that BMMSC-EVs exerted an immunosuppressive effect in GVHD mice, with effects on both cell survival and organ damage. The authors explained that this effect was due to the preservation of Tregs and the inhibition of functional differentiation of T cells from naive to effector type [114] (Table 1). In addition, BMMSC-EVs appeared to be potent inducers of Tregs, a feature that could have important therapeutic implications. Indeed, Del Fattore and coworkers (2015) found that BMMSC-EVs induced apoptosis of CD3 + cells and of the CD4 + subpopulation and increased the proliferation and apoptosis of Tregs [130].

In studies on myocardial infarction (MI), BMMSC-EVs improved cardiac function through stimulation of neo-angiogenesis and suppression of the inflammatory response, both in in vitro and in vivo experiments [115] (Table 1). Using cellular and animal models, Zou et al. (2019) confirmed the cardioprotection function of BMMSC-EVs [116] (Table 1). Similar findings have also been documented in liver diseases. Considering liver fibrosis, Rong and colleagues (2019) found for the first time that BMMSC-EVs effectively relieved disease through a Wnt/β-catenin pathway in in vitro experiments and in a CCl4-induced liver fibrosis model in rats [117] (Table 1). Instead, Gennai et al. demonstrated the protective effects of BMMSC-EVs in alleviating lung injury. They revealed that the intravenous administration of BMMSC-EVs had several beneficial effects including a decrease in organ weight in parallel with an increase in lung compliance [118]. From a mechanistic point of view, the authors, by using an approach including anti-CD44 antibodies, suggested that this molecule was responsible for the observed effects [118]. Recently, the efficacy of BMMSCs in the treatment of epidermolysis bullosa has been highlighted [131]. Since in this disease, patients lack the production of collagen type VII, which is essential for skin morphofunctional performance, BMMSC-EVs were shown by the authors to be able to restore the production of this collagen molecule in patient-specific fibroblasts, both via EV-dependent protein and mRNA transfer to target cells [119]. 

EVs, due to their characteristics and size, can be administered intravenously to patients and have the ability to pass through the blood–brain barrier. Therefore, they have been recently considered relevant in neurological damage and diseases. Chen et al. (2019) reported the administration of the BMMSC-EVs to mice with hippocampal damage following treatment of MSCs with a prostaglandin E2 receptor 4 (PGE2/EP4) antagonist. Specifically, the stimulation with PGE2/EP4 enriched the presence of several molecules in BMMSC-EVs, both growth factors (VEGF, BDNF) and cytokines [120]. The effect in vivo was related to the improvements in memory and learning tasks with respect to controls [120]. In addition, it has been suggested that stroke triggers the mobilization of BMMSC-EVs in patients with severe stroke [132]. BMMSC-EVs generated from MSCs cultured under conventional two-dimensional (2D) conditions or in three-dimensional (3D) collagen scaffolds, when administered to rats after traumatic brain injury (TBI), were capable of promoting functional recovery and neurovascular remodeling [121], as well as attenuating neuroinflammation conditions evoked by focal brain injury [133]. Importantly, some studies reported that EVs are the main mediators of MSC therapy in autism spectrum disorder (ASD). BTBR mice treated with BMMSC-EVs via intranasal administration showed significant improvements in autistic behavior, such as an increase in social interaction domain, ultrasonic communication, and repetitive behavior, when compared to mice transplanted with BMMSCs [122].

Regarding the MSC-EVs’ therapeutic benefits for cancer therapy, they can affect tumor cells and the TME by modifying tumor growth in different and sometimes opposite directions attributed to the heterogeneity of the same native MSCs populations [134]. More interestingly, BMMSC-EVs restrain the progression of lung cancer [123], gastric cancer [124], multiple myeloma [125], nasopharyngeal cancer [126], and other cancers (Table 1). On the other hand, BMMSC-EVs can also negatively regulate proliferation, migration, and tumorigenesis, as shown in prostate [127], colorectal [128], breast [129], and other cancers (Table 1).

In conclusion, the studies examined have highlighted the vast potential applications of BMMSC-EVs, demonstrating that the benefits of MSCs may either depend on the secretion of their EVs or can be effectively replicated by using EVs alone in a cell-free system.

### 4.2. Adipose Tissue MSC-Derived EVs

Adipose tissue is another reliable source of adult MSCs, which are able to secrete EVs (ADMSC-EVs) that have been demonstrated to be effective in a variety of diseases. As the parental cells do, ADMSC-EVs possess functions similar to BMMSC-EVs, such as anti-inflammatory activities and tissue repair properties. In studies of osteoarthritis, ADMSC-EVs stimulated with IL1β significantly reduced the production of inflammatory mediators, such as IL-6 and prostaglandin E2 [135] (Table 2).

As shown in Table 2, it has been reported that ADMSC-EVs exhibit anti-inflammatory properties in osteoarthritis by downregulating TNF-α, collagen II, and MMP activity. Of note, the anti-inflammatory IL-10 was upregulated, which also indicated that ADMSC-EVs could have anti-inflammatory effects [136]. In another work, ADMSC-EVs influenced T cells in several ways. First, they reduced their proliferation, but this effect was observed in parallel to a decreased differentiation of T cells [148]. ADMSC-EVs can reduce inflammation, as demonstrated recently [149]. The authors showed that molecules such as IL-10 and TGF-β were increased, as opposed to the reduction in the proinflammatory factors IL-6 and TNF-α in fibroblasts and THP-1 cells [149]. The promising indication of EVs in a chronic inflammatory setting was also confirmed when the authors treated EVs with melatonin, resulting in a switch to a M2 macrophage-mediated anti-inflammatory environment [149]. In addition, the anti-inflammatory effects of ADMSC-EVs were observed in allergic asthma. The authors showed that different molecules were influenced either locally or systemically. In particular, IL-5 decreased in the lungs together with IL-4 and IL-13 with further effects on CD3+CD4+ T cells, as well as on the lung mechanics [137] (Table 2). Meanwhile, it has also been shown that ADMSC-EVs have an immunosuppressive potential in atopic dermatitis [138], as well as favoring wound healing [139] (Table 2).

Previous studies reported that the administration of ADMSC-EVs exhibited a protective effect against MI [140], as well as vein graft intimal hyperplasia [141] (Table 2). Moreover, it has been found that co-transplantation of hypoxia-preconditioned ADMSC-EVs can effectively promote the survival of graft, neovascularization, inflammation, and hypoxia in a fat grafting animal model [150]. In another study, authors demonstrated that muscle tissue regeneration was stimulated the anti-inflammatory effects of EVs and soluble molecules, both in vitro and in vivo [142] (Table 2). Currently, growing interest is focused on the utilization of ADMSC-EVs to promote bone regeneration by improving the osteogenic differentiation of human BMMSCs, and authors well demonstrated the influence of EVs also in the prevention of senescence [143] (Table 2). Considering neurodegenerative diseases, a recent study showed a decrease in the amyloid beta (Aβ) peptide ratio (e.g., in the Aβ42/40) in neuronal cells, with a consequent increase in cell survival and also augmented neurite outgrowth of neuronal stem cells in Alzheimer’s disease [151].

In cancer, one of the experimental models studied was hepatocellular carcinoma. In this tumor, ADMSC-EVs increased response to pharmacological therapies due to their specific cargo molecules [144] (Table 2). It has further been observed that ADMSC-EVs attenuated cell proliferation and induced apoptosis in ovarian cancer [145] (Table 2). In another report, authors demonstrated that human dermal fibroblasts (HDFs) treated with ADMSC-EVs showed improvements in cell proliferation, migration, and tumorigenicity [146]. These findings suggest the ability of ADMSC-EVs to inhibit tumor formation. However, contrasting reports exist, in that ADMSC-EVs were shown to be able to interact with tumor cells, such as in breast cancer [146]. In fact, it was demonstrated that ADMSC-EVs promoted the migration and proliferation of MCF7 breast cancer cells, effects mediated by the upregulation of pathways regulating tumor development [147] (Table 2). We can conclude that several studies have illustrated the essential functions of ADMSC-EVs in many biological and pathological processes; however, it is clear that further works are needed to better understand the potential ADMSC-EV applications for the therapy of different diseases.

### 4.3. MSC-Derived EVs from Other Adult Sources

Even if the majority of studies regarding adult-derived EVs are centered on either BM or AD as the source of parental cells, it has been demonstrated that other sources may exist. As shown in Table 3, human corneal MSC-derived EVs (cMSC-EVs) could represent a novel therapeutic approach in the management of corneal wound healing disorders [152].

EVs derived from synovial mesenchymal stem cells (SMSC-EVs) may be an effective treatment for osteoarthritis [153] (Table 3). Recently, increasing evidence has shown that SMSC-EVs are able to influence chondrocytes in terms of their proliferative and migratory functions. Interestingly, the same authors also pointed out that extracellular matrix production was less changed in the in vivo model [153] (Table 3). Another tissue source for MSCs is DP. Tenuruma and colleagues revealed distinct transcriptomic signatures relative to neurogenesis and neural function in EVs derived from DP (DP-EVs), just like DP-MSCs, through in vitro experiments. These authors suggested that either DPMSC-EVs or DP-MSCs, due to their properties and expression panel, may be indicated to treat nervous system conditions, such as Alzheimer’s and Parkinson’s diseases [154] (Table 3). In addition, DPMSC-EVs are considered important for pulp/dentin and periodontal regeneration [155], as well as for immune-related pathologies [162]. Surprisingly, DPMSC-EVs could mitigate hematopoietic damage after radiation [156]. It has been observed that DPMSC-EVs performed comparable functions to epidermal growth factor (EGF) administration in their ability to regulate hematopoietic regeneration after radiation injury in vivo *and* promote proliferation and inhibit apoptosis of human umbilical vein endothelial cells (HUVECs) and FDC-P1 cells in vitro [156] (Table 3).

Human induced pluripotent stem cells (iPSCs) are obtained by the manipulation of somatic cells. Their features indicate that these cells can be used in personalized therapies since they can be directly derived from patients’ cells. Moreover, their molecular features somewhat resemble other stem cell types, such as embryonic stem cells (ESCs). Therefore, MSCs derived from iPSCs may represent another source of EVs for a cell-free therapeutic approach [163]. Comparative analyses between adult MSCs and human iPSC-MSCs showed that the latter bore a higher cell proliferation. Moreover, key features such as immunomodulation, the ability to secrete EVs, and general paracrine signaling were also strikingly higher in iPSC-MSCs [164]. In this regard, EVs secreted by iPSCs (iPSCMSC-EVs) possess a remarkable therapeutic effect in the treatment of osteoarthritis [157] and also reduce hepatic ischemia and reperfusion injury, promoting hepatocyte proliferation, both in vitro and in vivo [158] (Table 3). Another report showed that iPSCMSC-EVs can be indicated for intervertebral disc degeneration (IVDD). In fact, these EVs can promote the survival of resident cells in particular, as demonstrated by the authors, via activation of the Sirt6 pathway [159] (Table 3). Ultimately, iPSCMSC-EVs may represent a therapeutic strategy for cell-free skin regeneration approaches [160] (Table 3).

The liver is another organ that can be a source of adult-derived EVs. For example, it has been shown that EVs released by human liver stem-like cells (HLSCs) improved renal function by preventing and partially reverting fibrosis in diabetic nephropathy (DN), which may lead to end-stage chronic kidney disease (CKD) [110] (Table 3). Another interesting source of adult-derived EVs is the human endometrium, which contains specific MSCs. It has been shown that EVs released by endometrium-derived MSCs (EnMSCs) have a greater therapeutic potential when compared with cells derived from BM or AD [161] (Table 3). For example, EnMSC-EVs were capable of myocardial salvage and enhanced cardiac function after MI in the EnMSC-treatment groups, resulting in an increased expression of EVs [158]. Finally, we can also consider in this comparison the peripheral blood mononuclear cell (PBMC)-derived EVs for their ability to show cardioprotective effects in an in vivo model of acute MI [165].

## 5. Conclusions

In recent years, EVs from different sources of MSCs (i.e., BM, AD, and other sources) have experienced a revolutionary change in perspective, representing a promising carrier thanks to their better biocompatibility and intrinsic targeting ability. The reason for this lies in the fact that MSC-EVs appear to have immunomodulatory, anti-inflammatory, and regenerative properties that closely recapitulate the action of their parental cells. However, compared with adult MSCs, MSC-EVs offer several advantages, including lower immunogenicity and an improved safety profile, which make them a promising potential treatment in cell-free therapy approaches. In addition, studies are increasingly centered on extending the knowledge of the EV cargoes for the development of targeted therapies, allowing the mechanisms by which MSC-EVs exert their beneficial effect to be further detailed. In this regard, MSC-EVs may deliver functional proteins, RNAs, and other molecules to the recipient cells, thus regulating many biological processes (e.g., mainly via immunomodulation, tissue regeneration, antiapoptosis, and regulation of tumor progression), as well as mediating the intercellular communication and regulating gene expression. Nevertheless, although the current results are certainly encouraging, further research is still needed to optimize the applications of EVs as routine clinical tools. One of the main challenges to overcome is the optimization of methods for MSC-EV characterization for large-scale production using generalized and standardized protocols, since there are significant differences in the therapeutic activity of different MSC-EV entities. In addition, improvement is needed in MSC-EV targeting, which in parallel requires a better understanding of the disease setting before developing a treatment strategy based on EVs alone. Moreover, knowing the half-life and biodistribution of EVs once infused in the body is another aspect needing further investigation, since these features can also be affected by the employed administration route. In conclusion, on the basis of the promising results obtained to date, it is important to consider that the application of adult MSC-EVs in clinical practice requires further research focused on testing and extending the efficiency of this novel cell-free therapy, as it is influenced by the complexity of the composition of proteins, nucleic acids, and lipids which play a role in specific key roles. Nevertheless, several challenges need to be considered, such as safety/regulatory issues and technical aspects (e.g., harvesting procedure, cell expansion, and storage of the final cell yield), as well as the diversity of the donor subject as, in the light of a clinical translation, this will affect the effective ability of MSC-EVs against the same pathologies [166]. It follows that donor screening and the source of MSCs play an important role in determining the quantity, quality, and composition of EVs [167]. The use of EVs in clinical trials is well documented, with over 120 trials registered on the NIH clinicaltrials.org website to date. Indeed, only 15 use EVs derived from mesenchymal cells. In our opinion, given the large amount of data obtained from preclinical models in various diseases, an increase in the development of specific trials is further deserved in order to provide the due readout of the proposed effects in human therapies. In conclusion, these pieces of evidence support the concept that MSC-EVs may be able to interact with several cell types in a wide spectrum of diseases in different ways depending on their composition and properties. Nonetheless, the exact mechanism of in vivo action, long-term preservation, and possibility of delivery are not fully known and still deserve more research efforts.

## Figures and Tables

**Table 1:** Summary of adult human BMMSC-EV applications in various diseases.

Types of EVs	Diseases	Experimental Model	Biological Functions	Therapeutic Effects In Vivo	References
EVs	Osteoarthritis	In vitro	Inhibition of the proinflammatory interleukins (ILs) and of tumor necrosis factor-α (TNF-α)-mediated upregulation of cyclooxygenase 2 (COX2)		[106]
MVs	Renal injury	In vivo	Increased synthesis of hepatocyte growth factor (HGF) and macrophage-stimulating protein (MSP)	↑ tubuloepithelial regeneration ↑ proliferation	[107]
EVs	Renal ischemia–reperfusion injury	In vitro	Downregulation of apoptosis, cytoskeleton reorganization, and hypoxia		[108]
Exosomes	Renal fibrosis	In vitro and in vivo	Repression of transforming growth factor beta (TGF-βR1) protein expression	↓ kidney damage ↓ inflammation	[109]
EVs	Diabetic nephropathy	In vivo	Downregulation of Serpina1a, FAS ligand, C-C motif chemokine ligand 3 (CCL3), tissue inhibitor of metalloproteinases (TIMP1), matrix metalloproteinase 3 (MMP3), collagen I, and zinc finger protein SNAI1 (SNAI1)	↑ urinary volume ↑ water uptake ↑ albumin-to-creatinine ratio ↑ plasma creatinine	[110]
EVs	Inflammation	In vitro	Downregulation of the production of IL-23 and IL-22 via prostaglandin E_2_		[111]
Exosomes	Spinal cord injury	In vitro and in vivo	Decrease in inflammation and apoptosis, inhibition of phosphatase and tensin homolog (PTEN), and suppression of nuclear factor kappa-light-chain-enhancer of activated B cells (NF-κB) signal	↓ inflammation ↓ apoptosis	[112]
EVs	Patellar tendon injury	In vivo	Upregulation of collagen (COL)-1a1, scleraxis (SCX), tenomodulin (TNMD), and anti-inflammatory mediators (IL-10 and IL-4)	↓ inflammation ↓ apoptosis ↑ tendon stem/progenitor cells	[113]
EVs	Acute graft-versus-host disease	In vivo	Suppression of CD4+ and CD8+ T cells and of the functional differentiation of T cells from a naive to an effector phenotype, and preservation CD4 + CD25 + Foxp3+ regulatory T-cell populations	↓ CD4+ and CD8+ T cells ↓ differentiation of T cells ↑ naive T populations	[114]
EVs	Myocardial infarction	In vitro and in vivo	Suppression of the inflammation response and promotion of blood vessel formation	↑ blood flow ↑ cardiac systolic ↑ cardiac diastolic	[115]
Exosomes	Myocardial infarction	In vitro and in vivo	Downregulation of apoptosis via regulating B-cell lymphoma-associated X (Bax)	↑ cardiac function ↓ apoptosis	[116]
Exosomes	Liver fibrosis	In vitro and in vivo	Downregulation of the expression of peroxisome proliferator-activated receptor gamma (PPARγ), Wnt3a, Wnt10b, β-catenin in the Wnt signaling pathway and, consequently, inhibition of downstream gene expression WNT1-inducible-signaling pathway protein 1 (WISP1) and cyclin D1	↓ fibrosis ↓ inflammation ↑ liver function ↑ hepatocyte regeneration	[117]
Microvesicles	Pulmonary edema for transplantation	Ex vivo	Reduction in syndecan-1 levels and increase in angiopoietin 1 (Ang1) levels		[118]
EVs	Dystrophic epidermolysis bullosa	In vitro	Increase and transfer of type VII collagen and mRNA to recipient cell		[119]
EVs	Hippocampal CA1 neuron damage	In vivo	Increased levels of anti-inflammatory cytokines and neuron support proteins including IL-2, IL-10, CCL5, vascular endothelial growth factor (VEGF), and brain-derived neurotrophic factor (BDNF)	↓ astrogliosis ↓ inflammation ↓ microglial infiltration	[120]
Exosomes	Traumatic brain injury	In vivo	Increased angiogenesis and neurogenesis pathway	↑ cognitive function ↑ sensorimotor function ↓ neuroinflammation	[121]
Exosomes	Autism spectrum disorders	In vivo	Regulation of the cell cycle progression, proliferation, and modulation of angiogenesis	↑ in all ASD-like symptoms	[122]
EVs	Lung cancer	In vitro	Activation of protein kinase B (PKB or Akt) and signal transducer and activator of transcription 3 (STAT3), upregulation of cell mobility by reversion-inducing cysteine-rich protein with kazal motifs (RECK) targeting, and induction of macrophage M2 polarization		[123]
Exosomes	Gastric cancer	In vitro	Upregulation of the expression of MMP-14		[124]
Exosomes	Multiple myeloma	In vitro and in vivo	Increased expression of oncogenic proteins, cytokines, and protein kinases	↑ tumor growth	[125]
Exosomes	Nasopharyngeal cancer	In vitro and in vivo	Activation of extracellular signal-regulated kinases (ERKs) and p38 MAPK pathway	↑ tumor formation ↑ tumor growth	[126]
Exosomes	Prostate cancer	In vitro and in vivo	Overexpression of miR-99b-5p and reduction in expression levels of insulin-like growth factor 1 receptor (IGF1R)	↓ tumor growth	[127]
EVs	Colorectal cancer	In vitro and in vivo	Downregulation of activating transcription factor 3 (ATF3), and activation of the AKT signaling	↓ tumor growth ↓ immune escape	[128]
EVs	Breast cancer	In vitro	Inhibition of myristoylated alanine-rich c-kinase substrate (MARCKS), resulting in reduced cell cycling and motility		[129]

Upward arrow means increase, Downward arrow means decrease.

**Table 2:** Summary of human ADMSC-EV applications in various diseases.

Types of EVs	Diseases	Experimental Model	Biological Functions	Therapeutic Effects In Vivo	References
Microvesicles and exosomes	Osteoarthritis	In vitro	Downregulation of IL-6 and PGE2. Reduction in oxidative stress		[135]
Microvesicles and exosomes	Osteoarthritis	In vitro	Downregulation of PGE2, TNF-α, NO, collagen II. Reduction in MMP activity. Upregulation of IL-10		[136]
EVs	Allergic asthma	In vivo	Reduction in collagen fiber deposition, TGF-β and IL-5 levels, total cell count, and the percentage of CD3 + CD4 + T cells in the thymus	↓ static lung elastance ↓ Tregs ↓ CD3+CD4+ T cells	[137]
Exosomes	Atopic dermatitis	In vivo	Reduction in serum levels of IgE and eosinophils		[138]
Exosomes	Wound healing	In vitro and in vivo	Increased gene expression of N-cadherin, cyclin-1, proliferating cell nuclear antigen (PCNA), and collagen I and III in the early stage; inhibition of the collagen expression during the late stage	↑ collagen I and III ↓ scar formation	[139]
Exosomes	Myocardial infarction	In vitro and in vivo	Inhibition of apoptosis and suppression of inflammatory response, promotion of angiogenesis	↓ apoptosis ↑ angiogenesis	[140]
EVs	Vein graft intimal hyperplasia	In vitro and in vivo	Decreased macrophage infiltration, attenuation of inflammatory cytokine expression, and reduction in activation of MAPK and phosphatidylinositol-3 kinase (PI3K) signaling pathways	↓ inflammation ↓ intimal hyperplasia ↓ macrophage infiltration	[141]
EVs	Muscle generation	In vitro and in vivo	Regulation of immune system processes (e.g., innate immune response) and immunological development	↑ cross-sectional area of new muscle fibers ↓ macrophage infiltration	[142]
Exosomes	Bone regeneration	In vitro and in vivo	Inhibition of insulin-like growth factor binding protein 3 (IGFBP3)	↑ osteogenic differentiation	[143]
Exosomes	Hepatocellular carcinoma	In vitro and in vivo	Induction of apoptosis and cell cycle arrest	↑ antitumor activity of multikinase inhibitor	[144]
Exosomes	Ovarian cancer	In vitro	Regulation of cell cycle progression, cytokine and cytokine-receptor expression, and cancer survival by downregulation of different CDKs		[145]
Exosomes	Ischemic wounds	In vitro and in vivo	Increase in the expression of lncRNA metastasis-associated lung adenocarcinoma transcript 1 (MALAT1)	↑ healing of ischemic wounds ↑ human dermal fibroblast migration	[146]
Exosomes	Breast cancer	In vitro	Upregulation of Wnt signaling pathway		[147]

Upward arrow means increase, Downward arrow means decrease.

**Table 3:** Summary of applications of other human adult MSC-derived EVs in various diseases.

EV Cell Origin	Types of EVs	Diseases	Experimental Model	Biological Functions	References
cMSC-EVs	Exosomes	Nonhealing corneal wounds	In vitro	Increased migration and proliferation of the epithelial cells	[152]
SMSC-EVs	Exosomes	Osteoarthritis	In vitro and in vivo	Activation of YAP through alternative Wnt signaling pathways, and suppression of the expression of SOX9	[153]
DPMSC-EVs	EVs	Neurological disorders	In vitro	Regeneration or improved functions of neural system	[154]
DPMSC-EVs	Exosomes	Periodontal regeneration	In vitro and in vivo	Suppression of inflammation by facilitating the conversion of macrophages from a proinflammatory phenotype to an anti-inflammatory phenotype	[155]
DPMSC-EVs	EVs	Hematopoietic regeneration	In vitro and in vivo	Favoring regeneration of specific hematopoietic cell lineages, such as CD13/14+, CD19+, CD3+, CD41+, and CD71/glycophorin A+	[156]
iPSCMSC-EVs	Exosomes	Osteoarthritis	In vitro	Stimulating chondrocyte migration and proliferation	[157]
iPSCMSC-EVs	Exosomes	Hepatic ischemia–reperfusion injury	In vitro and in vivo	Suppression of hepatocyte necrosis, sinusoidal congestion, and increased activity of sphingosine kinase and synthesis of sphingosine-1-phosphate (S1P)	[158]
iPSCMSC-EVs	EVs	Intervertebral disc degeneration	In vivo	Increased cAMP concentrations by targeting phosphodiesterase 4D (PDE4D) and activating Sirt6 pathway	[159]
iPSCMSC-EVs	Exosomes	Skin wound	In vitro and in vivo	Increase in cell growth, gene expression, and ERK1/2 phosphorylation	[160]
HLSC-EVs	EVs	Diabetic nephropathy	In vivo	Downregulation of Serpin 1a, FAS ligand, CCL3, TIMP1, MMP3, collagen I, and SNAI1	[110]
EVs derived from EnMSCs	Exosomes	Myocardial infarction	In vitro and in vivo	Positive regulation of the target PTEN-Akt survival pathway	[161]

cMSC-EVs, human corneal MSC-derived EVs; DP MSC-EVs, dental pulp MSC-derived EVs; EnMSCs, endometrium-derived MSCs; HLSC-EVs, human liver stem-like cell MSC-derived EVs; iPSC-EVs, human induced pluripotent stem cell MSC-derived EVs; PBMCs, peripheral blood mononuclear cells.

## Abbreviations

ACHE, acetylcholinesterase; AD, adipose tissue; Ang1, angiopoietin 1; Apaf 1, apoptotic protease activating factor-1; ASD, autism spectrum disorder; ATF3, activating transcription factor 3; ATP, adenosine triphosphate; Akt, protein kinase B; Aβ, amyloid beta; BDNF, brain-derived neurotrophic factor; BM, bone marrow; CD, cluster of differentiation; CCL3, C-C motif chemokine ligand 3; CDK, cyclin-dependent kinase; CFU-F, colony-forming unit fibroblastic; COX2, cyclooxygenase 2; DP, dental pulp; ECM, extracellular matrix; EE, early endosome; ER, endoplasmic reticulum; ERK, extracellular signal-regulated kinase; ESCRT, endosomal sorting complex required for transport; ESCs, embryonic stem cells; EVs, extracellular vesicles; GVHD, graft versus host disease; HLSCs, human liver stem-like cells; HUVEC, human umbilical vein endothelial cell; IGF1R, insulin-like growth factor 1 receptor; IGFBP3, insulin-like growth factor-binding protein 3; lnc-RNA, noncoding RNA; IVDD, intervertebral disc degeneration; iPSC, induced pluripotent stem cell; IL, interleukin; ILVs, intraluminal vesicles; ISVE, International Society for Extracellular Vesicles; MALAT1, metastasis-associated lung adenocarcinoma transcript 1; MAPK, mitogen-activated protein kinase; MARCKS, myristoylated alanine-rich c-kinase substrate; MHC, major histocompatibility complex; MI, myocardial infarction; miRNA, microRNA; MMP, matrix metalloproteinase; mRNA, messenger RNA; MSCs, mesenchymal stromal cells; MVs, microvesicles; MVBs, multivesicular bodies; mTOR, mechanistic target of rapamycin; NF-κB, nuclear factor kappa-light-chain-enhancer of activated B cells; NPCs, senescent nucleus pulposus cells; PBMC, peripheral blood mononuclear cell; PCNA, proliferating cell nuclear antigen; PDE4D, phosphodiesterase 4D; PIP, phosphoinositide; PGE2, prostaglandin E2; PI3K, phosphatidylinositol-3 kinase; PPARγ, peroxisome proliferator-activated receptor gamma; PTEN, phosphatase and tensin homolog; RECK, reversion-inducing cysteine-rich protein with kazal motifs; SCX, scleraxis; SNAI1, zinc finger protein SNAI1; STAT3, signal transducer and activator of transcription 3; TBI, traumatic brain injury; TGF-β, transforming growth factor beta; TGN, trans-Golgi network; TIMP 1, tissue inhibitor of metalloproteinases 1; TNMD, tenomodulin; TNF-α, tumor necrosis factor-α; Treg, regulatory T cell; VEGF, vascular endothelial growth factor; VPSA, vacuolar protein sorting-associated protein A; VTA1, vacuolar protein sorting-associated protein VTA1 homolog; WISP1, WNT1-inducible-signaling pathway protein 1.
